# Isorhapontigenin ameliorates cerebral ischemia/reperfusion injury via modulating Kinase Cε/Nrf2/HO‐1 signaling pathway

**DOI:** 10.1002/brb3.2143

**Published:** 2021-06-08

**Authors:** Zhe Xue, Kai Zhao, Zhenghui Sun, Chen Wu, Bowen Yu, Dongsheng Kong, Bainan Xu

**Affiliations:** ^1^ Department of Neurosurgery Chinese PLA General Hospital Beijing China; ^2^ Department of Neurosurgery Hainan Hospital of Chinese PLA General Hospital Beijing China

**Keywords:** cerebral ischemia, HO‐1, isorhapontigenin, Nrf2, oxidative stress, PKCε, reperfusion

## Abstract

**Background:**

Isorhapontigenin (ISO) has been shown to have antioxidant activity. This study aimed to investigate the antioxidant effects of ISO on cerebral ischemia/reperfusion (I/R) injury and its possible molecular mechanisms.

**Methods:**

Focal cerebral ischemia‐reperfusion injury (MCAO/R) model and primary cortical neurons were established an oxygen‐glucose deprivation (OGD / R) injury model. After 24 hr of reperfusion, the neurological deficits of the rats were analyzed and HE staining was performed, and the infarct volume was calculated by TTC staining. In addition, the reactive oxygen species (ROS) in rat brain tissue, the content of 4‐Hydroxynonenal (4‐HNE), and 8‐hydroxy2deoxyguanosine (8‐OHdG) were detected. Neuronal cell viability was determined by MTT assay. Western blot analysis was determined for protein expression.

**Results:**

ISO treatment significantly improved neurological scores, reduced infarct volume, necrotic neurons, ROS production, 4‐HNE, and 8‐OHdG levels. At the same time, ISO significantly increased the expression of Nrf2 and HO‐1. The neuroprotective effects of ISO can be eliminated by knocking down Nrf2 and HO‐1. In addition, knockdown of the PKCε blocked ISO‐induced nuclear Nfr2, HO‐1 expression.

**Conclusion:**

ISO protected against oxidative damage induced by brain I/R, and its neuroprotective mechanism may be related to the PKCε/Nrf2/HO‐1 pathway.

## Graphical abstract

ISO attenuated cerebral I/R injury in rats by activating the PKCε/Nrf2/HO‐1 signaling pathway. As a survival signaling pathway, PKCε/Nrf2/HO‐1 signaling pathway may become a therapeutic target for ischemic cerebrovascular diseases in the future.

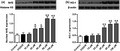

## 1 BACKGROUND

Cerebrovascular diseases are common in clinics and have high morbidity, which seriously threatens patients’ life safety (Wang et al., 2018). Among them, ischemic cerebrovascular disease is the leading cause of sudden death caused by cerebrovascular diseases (Che et al., 2017; Shih‐Wei & Cheng, 2018). At present, early return of blood supply to the ischemic area is still the treatment foundation (Liao et al., 2018; Zuo et al., 2017). However, reperfusion could lead to injury to the ischemic areas, further aggravating brain tissue damage and resulting in more severe brain dysfunction (Margherita et al., 2017; Xiaolong et al., 2020).

The development of ischemic reperfusion (I/R) injury is a highly complicated pathophysiological process and involves many factors (Wang et al., 2017; Zhang et al., 2017). Although our understanding of brain I/R injury has gradually deepened, its specific mechanism has not been fully elucidated. Scientists often utilize rats with middle cerebral artery occlusion (MCAO) as the experimental acute cerebral ischemia model simulated human ischemic‐hypoxic encephalopathy (Sadana et al., 2015) and believed that the major causes for cerebral I/R injury after ischemia and hypoxia of the cerebral nerve cells are apoptosis, impaired energy metabolism, production of various apoptosis stimulating factors, nitric oxide, and excitatory amino acids (Hu et al., 2017; Zheng et al., 2016). Studies have shown that the highly conserved protein kinase Cε (PKC‐ε) is involved in many pathological processes such as oxidative stress, thrombosis, and fibrosis (Guo et al., 2017; Horst et al., 2017; Yang et al., 2019). In many oxidative stress animal models, PKCε is closely associated with lung injury, either by drug blockade or by gene knockout (Liu, et al., 2017; Yadav et al., 2018). The cytoplasm‐localized transcription factor Nrf2 is closely related to oxidative stress (Je & Lee, 2015; Zhu et al., 2017) and induces transcription of many ARE‐dependent target genes (Abiko et al., 2014; Zhong & Kowluru, 2012), including HO‐1. HO‐1 expression is increased under oxidative stress and plays an antioxidant role in the pathological development of various diseases (Dias‐Teixeira et al., 2017). Whether the PKC‐ε/Nrf2/HO‐1 pathway affects I/R injury has not been reported.

Isorhapontigenin (ISO) is a known stilbene component extracted from the traditional Chinese medicine *Gnetum cleistostachyum* (Jiang et al., 2018; Yeo et al., 2017). ISO has anti‐inflammation and anti‐oxidation functions (Dai et al., 2018; Zhu, Zhu, et al., 2017). Its protective effect on cardiovascular diseases is mainly achieved through anti‐oxidation, scavenging‐free radicals, anti‐inflammation, regulating blood lipids, anti‐proliferation, and affecting cell signal transduction (Liu, et al., 2017; Yang, et al., 2017). However, the molecular mechanisms underlying the action of ISO on cerebrovascular diseases have not been fully revealed. In this study, the rat MACO model was used to investigate further whether ISO exerts its neuroprotective effect via PKCε/Nrf2/HO‐1 signaling pathway and explore its possible mechanisms with the hope to provide new research directions for identifying new drugs for cerebral ischemic injury.

## 2 METHODS

### 2.1 Chemicals and reagents

**FIGURE 1:**
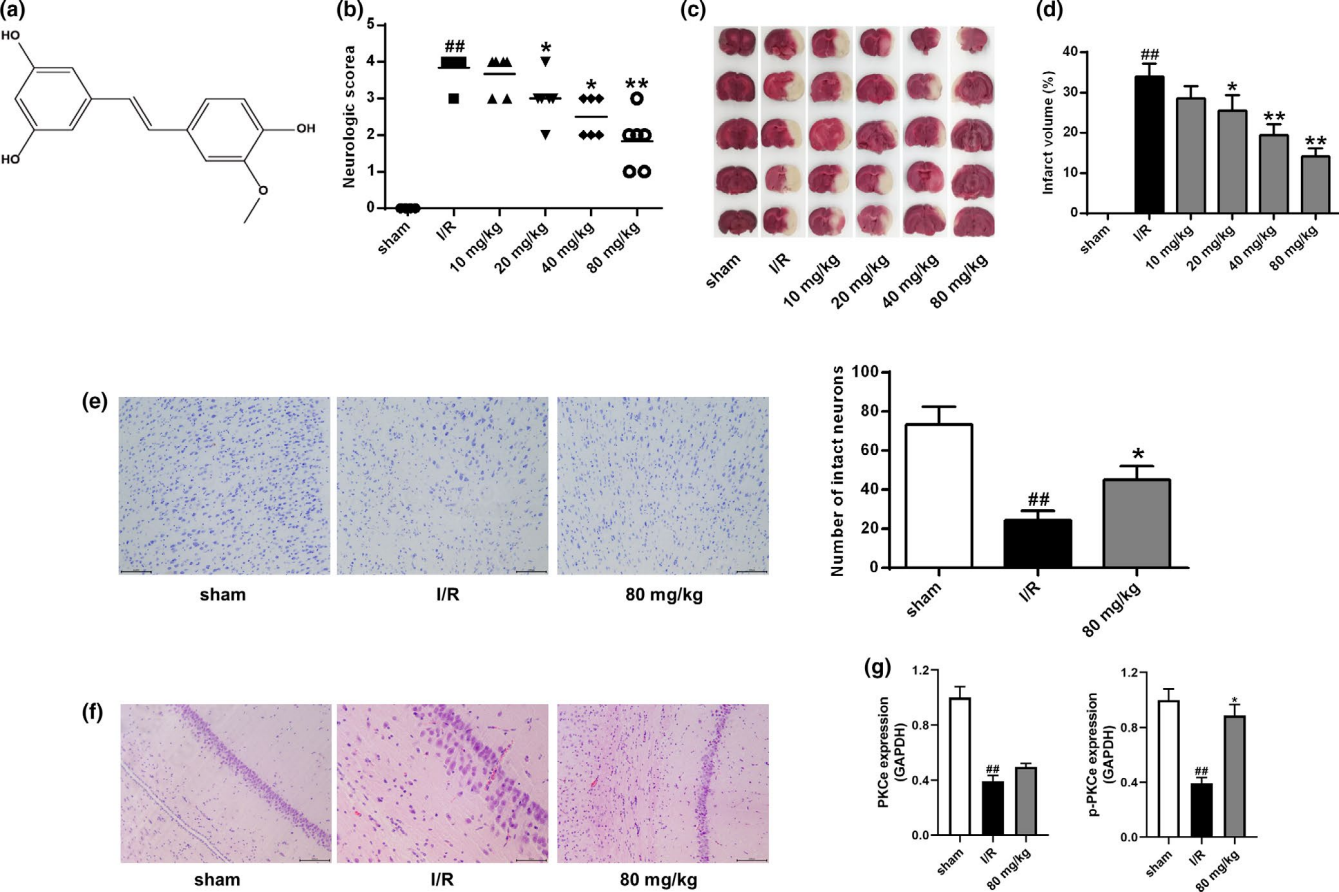
ISO protected the brain from I/R damage. (a) The chemical structure of ISO. (b) Neurological deficit score. (c) Cerebral infarction in rat brain. (d) Percentage of infarct volume in the brain. (e) Nissl staining to assess rat neuronal damage. (f) H&E staining to assess rat neuronal damage. (g) PKCε and phosphorylated PKCε levels in the brain. Scale bar =100 μm (×200 times). *n* = 6. ## *p* < .01 versus the sham group; *p < .05 and **p < .01 versus the I/R group

ISO (purity ≥98%) with the structure shown in Figure 1a was purchased from Sigma Chemical Co. 4‐HNE and 8‐OHdG were purchased from Cell Biolabs.

### 2.2 Experimental animals

Wistar rats, both male and female, weighing 185 to 225 g, were provided by the Chinese PLA General Hospital’ Experimental Animal Center. Animal experiments were approved by the Animal Ethics Committee following the guidelines for the care and use of laboratory animals.

### 2.3 Focal cerebral ischemia/reperfusion (I/R) model

A total of 15 Wistar rats were randomly divided into three groups according to body weight. After 2 hr of ischemic attack, ISO was slowly injected intraperitoneally into the rats. After the last administration, rats were put under anesthesia by intraperitoneal injection of pentobarbital (40 mg/kg) and the MCAO model was prepared as reported previously (Janyou et al., 2017). The nylon thread was slowly advanced into the cranial artery through the main carotid artery incision to reach the cerebral artery. After 2 hr, the artery stump was tightened, and the ischemia‐reperfusion injury model was completed.

### 2.4 Assessment of infarct volume and neurological deficits

After 24 hr of I/R injury, the rats were decapitated, and the olfactory bulb, cerebellum, and low brainstem were removed and snap‐frozen. The brain tissue was sliced and stained with triphenyl tetrazolium chloride (TTC) for 30 min with white staining for the infarct brain and red for the normal brain. After pictures were taken, an image analysis system was used to measure the area of cerebral infarction. After 24 hr of cerebral ischemia‐reperfusion, rat neurological deficits were scored according to the 5‐point standard reported previously (Kewei et al., 2017).

### 2.5 Nissl staining

After 24 hr of I/R injury, rats were decapitated and perfused with 4% paraformaldehyde. The brain tissue was fixed in a fixing agent for 1 day. Then, the brain sections were subjected to Nissl staining, and the neuronal death was analyzed under a light microscope.

### 2.6 H&E staining

After deep anesthesia, rats were perfused with normal saline and 40 g/L paraformaldehyde fixative and stored in 200 g/L sucrose overnight. Coronal sections of 30‐μm brain tissue were made at 2 mm after optic chiasm by cryomicrotome and 10‐ to 15‐μm sections of adjacent tissues were made. These sections were then subjected to H&E staining.

### 2.7 ROS measurement

ROS were detected using 2',7',‐dichlorofluorescein diacetate (DCFH‐DA) following the manufacturer's instruction, and fluorescence at 525 nm was read using a fluorescent plate reader.

### 2.8 4‐HNE and 8‐OHdG measurement

8‐hydroxydeoxyguanosine (8‐OHdG) and 4‐hydroxynonenal (4‐HNE) levels in the cortex of each group (*n* = 5) were detected by immunohistochemistry. In brief, frozen brain sections were incubated in 0.3% H_2_O_2_/methanol. After blocked in a mouse IgG blocking solution, they were incubated at 4ºC with antibodies against 4‐HNE (1:50; R&D Systems) and 8‐OHdG (1:20; R&D Systems), respectively. Immunoreactivity was visualized by a biotinylated secondary antibody, avidin‐biotin‐peroxidase complex, and DAB.

### 2.9 Cell culture

The human neuroblastoma cell line SH‐SY5Y was obtained from the American Type Culture Collection (ATCC), subcultured in DMEM medium containing 10% fetal bovine serum (FBS, GIBCO) and differentiate into neuron‐like cells after 10‐μM retinoic acid stimulation.

### 2.10 Treatment and oxygen deprivation/reperfusion (OGD/R)

The SH‐SY5Y cells were cultured for 5–7 days. For treatment, cells in the model group were incubated in sugar‐free neurobasal medium containing the corresponding drugs in an incubator supplemented with 5% CO_2_ and 1% O_2_ at 37°C for 1.5 hr and then in neurobasal containing 2% B27 medium for additional 24 hr.

### 2.11 Cell viability determination

SH‐SY5Y cells were seeded in 96‐well plates. After treatment, cells were incubated with 5 μl of 5 g/L MTT phosphate buffer. After discarding the supernatants to preserve the bottom sediment, 100 μl DMSO was added to each well, and the absorbance of each well was measured at 570 nm. The relative cell viability of each group was determined by comparison to the control.

### 2.12 Transient transfection of small interference RNA (siRNA)

PKCε, Nrf2, and HO‐1 siRNAs were designed and synthesized by Jima Biotechnology, Inc. For targeted specific transient transfection, siRNAs were transiently transfected into differentiated SH‐SY5Y cells by RNAiMax and Lipofectamine 3000 with Plus Reagent (Thermo Fisher Scientific). After 48 hr, cells were treated with ISO and subjected to a 2 hr OGD challenge followed by reoxygenation for 24 hr. Transfection efficiency was determined by Western blot.

### 2.13 Western blot analysis

24 hr after MCAO/R or OGD/R, cytoplasm and nuclear proteins were extracted using Nuclear/Cytosol Fractionation Kit (BioVision) and quantified using the BCA Protein Assay Kit. Proteins were separated by sodium dodecyl sulphate–polyacrylamide gel electrophoresis (SDS‐PAGE) and transferred by electrophoresis onto PVDF membranes. The membranes were incubated with antibodies against PKCε (1:500), Nrf2 (1:500), HO‐1 (1:500), β‐actin (1:1,000), histone H3 (1:1,000), and GAPDH (1:1,000) (Abcam) overnight. After that, the membranes were incubated with 1:5,000 labeled anti‐rabbit secondary antibody for 1 hr and labeled proteins were visualized and quantified as reported previously (Kopec et al., 2017).

### 2.14 Statistical analyses

All data were analyzed by SPSS version 19.0 statistical software and shown as mean ±standard deviation (*SD*). Multigroup data analysis was analyzed using one‐way ANOVA followed by LSD test *p* < .05 indicates significant difference.

## 3 RESULTS

### 3.1 ISO protected brain against I/R injury

Neurological deficits were scored 24 hr after reperfusion. The results showed that neurological deficit scores were significantly increased in the I/R group compared with the sham group (Figure 1b, *p* < .01), and this increase was attenuated by ISO in a dose‐dependent manner (*p* < .05, *p* < .01). TTC staining for cerebral infarct size showed that a significant increase in the percentage of infarct volume in the I/R group compared with the sham group (*p* < .01), and this increase was also significantly reduced by ISO treatment in a dose‐dependent manner (Figure 1c,d, *p* < .05, *p* < .01).

Neuron morphologic observation by Nissl staining (Figure 1e) showed abnormal hippocampus structures and condensed and deeply stained nuclei in I/R group but intact and normal hippocampus structures and cell membrane in the sham group. Moreover, the number of hippocampal neurons was significantly decreased in the I/R group compared with the sham group (*p* < .01). By contrast, the pyramidal cells in the hippocampus were more regular and arranged in a more precise manner in the ISO group and the number of hippocampal neurons in the ISO group was also significantly increased compared with the I/R group (*p* < .05). Neuron morphologic observation by H&E staining (Figure 1f) also showed more disorderly aligned neurons in the I/R group than in sham and ISO groups and better aligned neurons in the ISO group than in the I/R group. These results indicated that ISO reduces brain I/R damage. Moreover, PKCε was down‐regulated in rats in the I/R group and ISO treatment attenuated the decrease of PKCε protein.

### 3.2 Effects of ISO on oxidative damage

**FIGURE 2:**
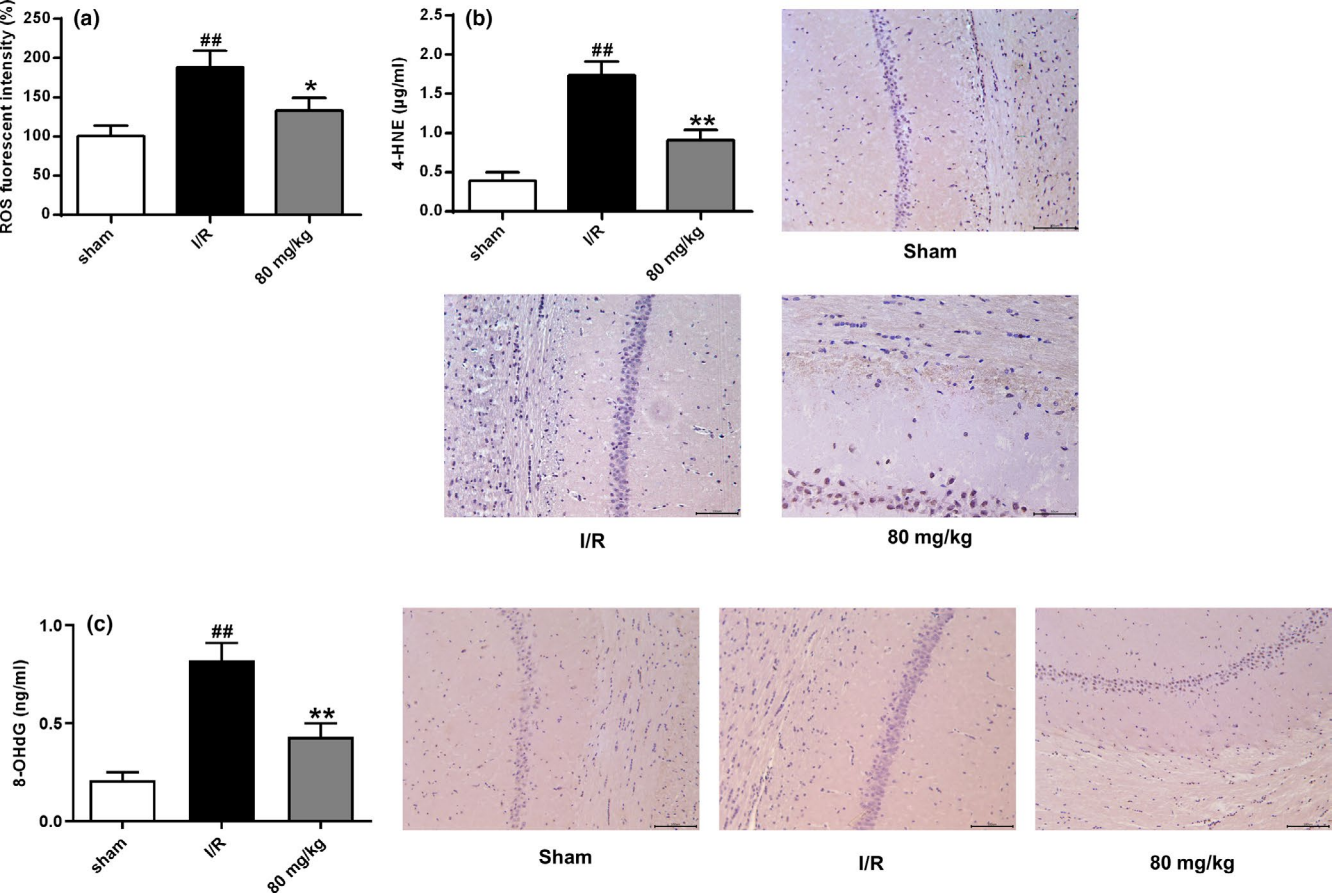
ISO reduced oxidative damage. (a) ROS levels. (b) 4‐HNE content. (c) 8‐OHdG content. *n* = 6. ## *p* < .01 versus the sham group; *p < .05 and **p < .01 versus the I/R group

As shown in Figure 2a, compared the sham group, the amount of ROS in the I/R group was significantly increased (*p* < .01). Compared with the I/R group, the amount of ROS after ISO treatment was significantly reduced (*p* < .05). As shown in Figures 2, 4b,c, 4‐HNE and 8‐OHdG levels were significantly higher in the I/R group than in the sham group (*p* < .01) but lower in the ISO group than in the I/R group (*p* < .01). These data indicated that ISO treatment partially reversed oxidative brain damage by I/R.

**FIGURE 3:**
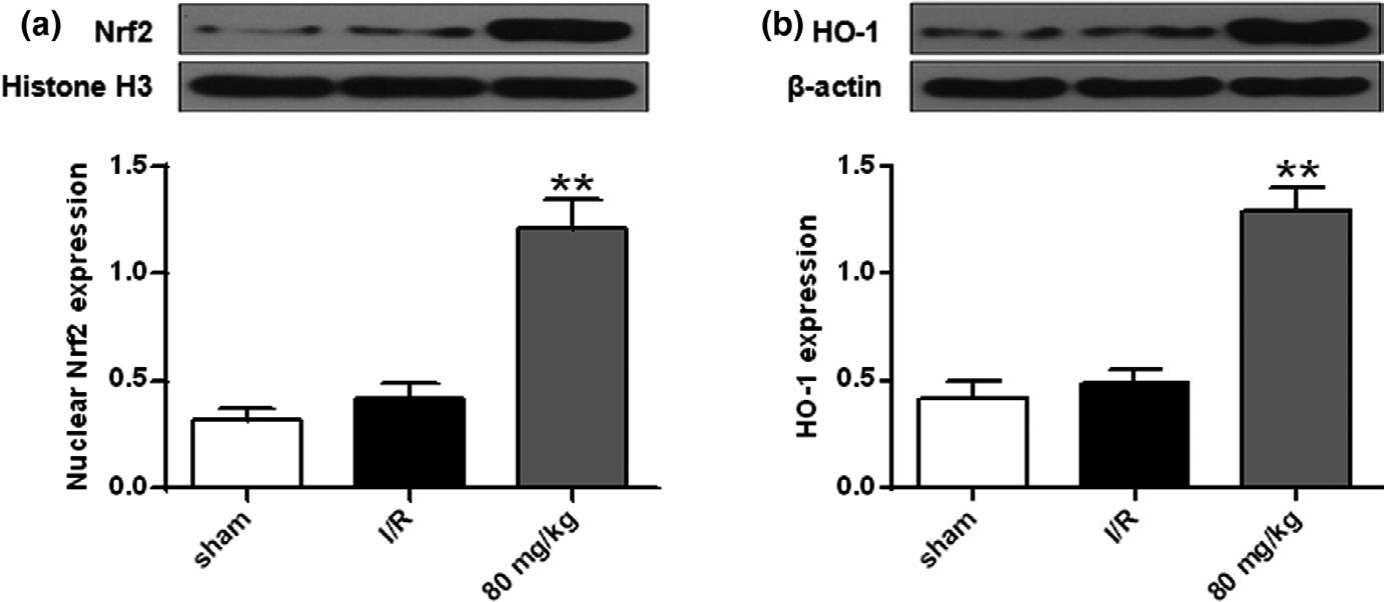
Protein expression levels of Nrf2 (a) and HO‐1 (b). *n* = 6. ** *p* < .01 versus the sham group

**FIGURE 4:**
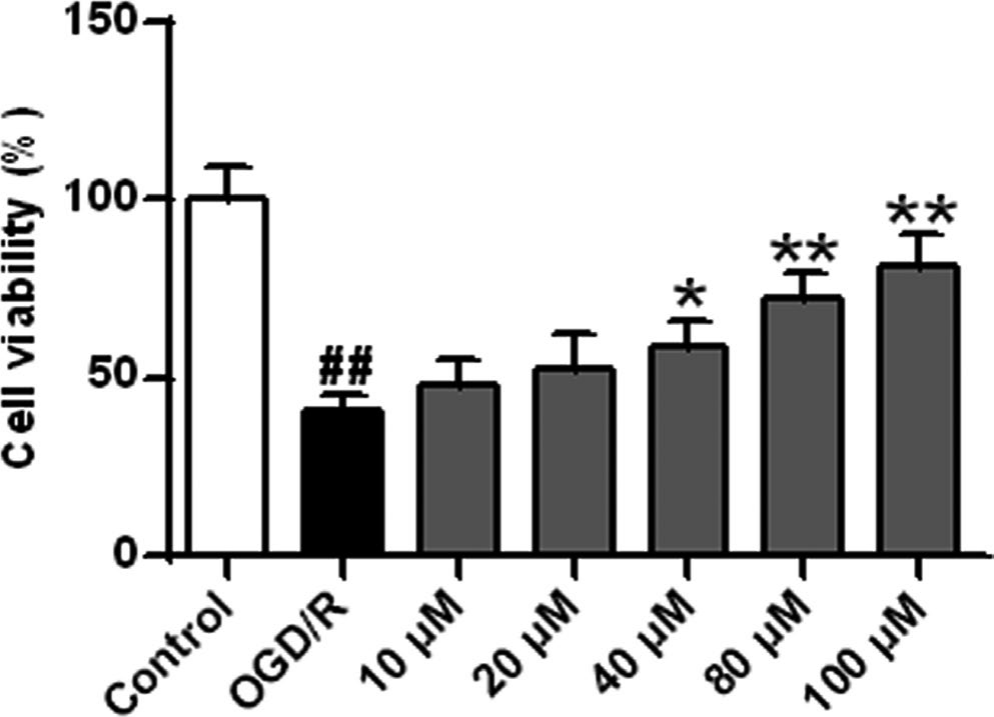
Cell viability determined. *n* = 6. ^##^ *p* < .01 versus the sham group; *p < .05 and **p < .01 versus the OGD/R group

### 3.3 Effect of ISO on Nrf2 and HO‐1 expression

As shown in Figure 3a,b compared with the sham group, Nrf2 and cytoplasmic HO‐1 protein levels in the I/R group were slightly increased (*p* > .05). Compared with the I/R group, nuclear Nrf2 and cytoplasmic HO‐1 protein levels were significantly increased after ISO treatment (*p* < .01). These data indicated that ISO reduced oxidative brain damage by I/R (*p* < .01).

### 3.4 ISO protected cells against OGD/R injury

The neuroprotective effects of ISO on differentiated SH‐SY5Y cells at 48 hr were further analyzed. As shown in Figure 4, compared with the control group, cell viability was significantly lower in the OGD/R group (*p* < .01). Compared with the OGD/R group, cell viability was increased gradually after ISO treatment in a dose‐dependent manner (*p* < .05, *p* < .01). These results indicated that ISO protected differentiated SH‐SY5Y cells from OGD/R‐induced damage.

### 3.5 ISO promoted Nrf2 nuclear translocation and HO‐1 up‐regulation in vitro

**FIGURE 5:**
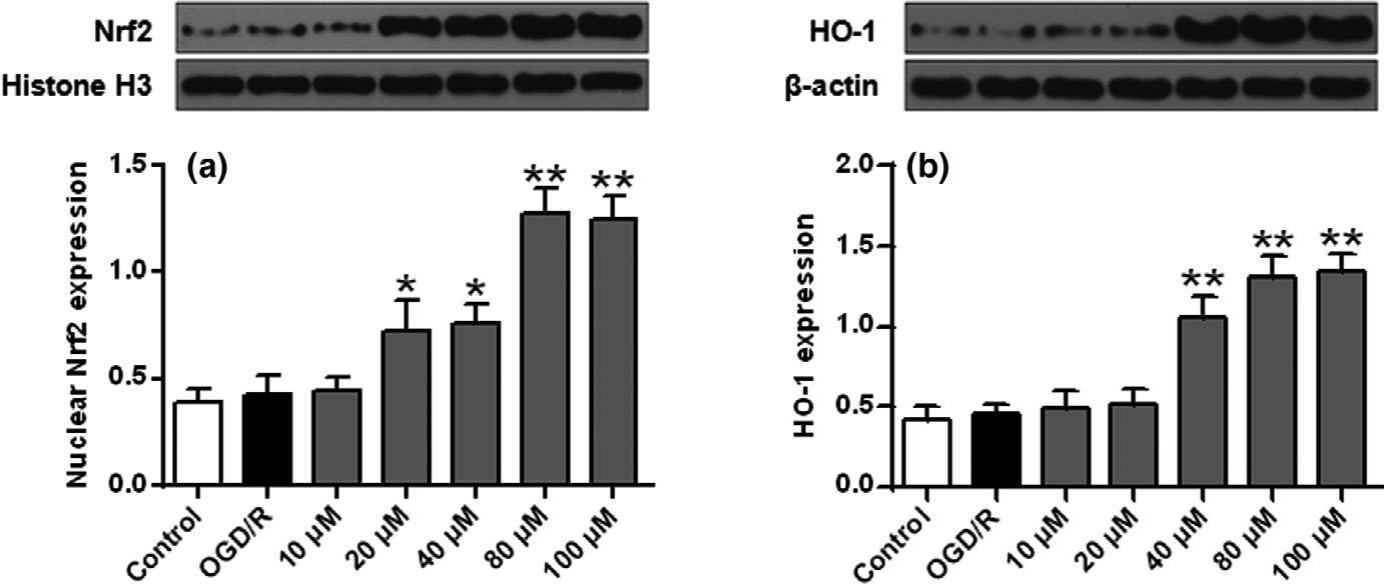
ISO’s effects on protein expression of Nrf2 (a) and HO‐1 (b) in SH‐SY5Y cells. *n* = 6. * *p* < .05 and **p < .01 versus the OGD/R group

As shown in Figure 5a,b, compared with the control cells, nuclear Nrf2 and cytoplasmic HO‐1 protein expression were slightly increased in the OGD/R group (*p* > .05). Compared with the OGD/R group, nuclear Nrf2 and cytoplasmic HO‐1 protein levels were significantly increased after ISO treatment (*p* < .05, *p* < .01).

### 3.6 The neuroprotection of ISO involved the Nrf2/HO‐1 pathway

**FIGURE 6:**
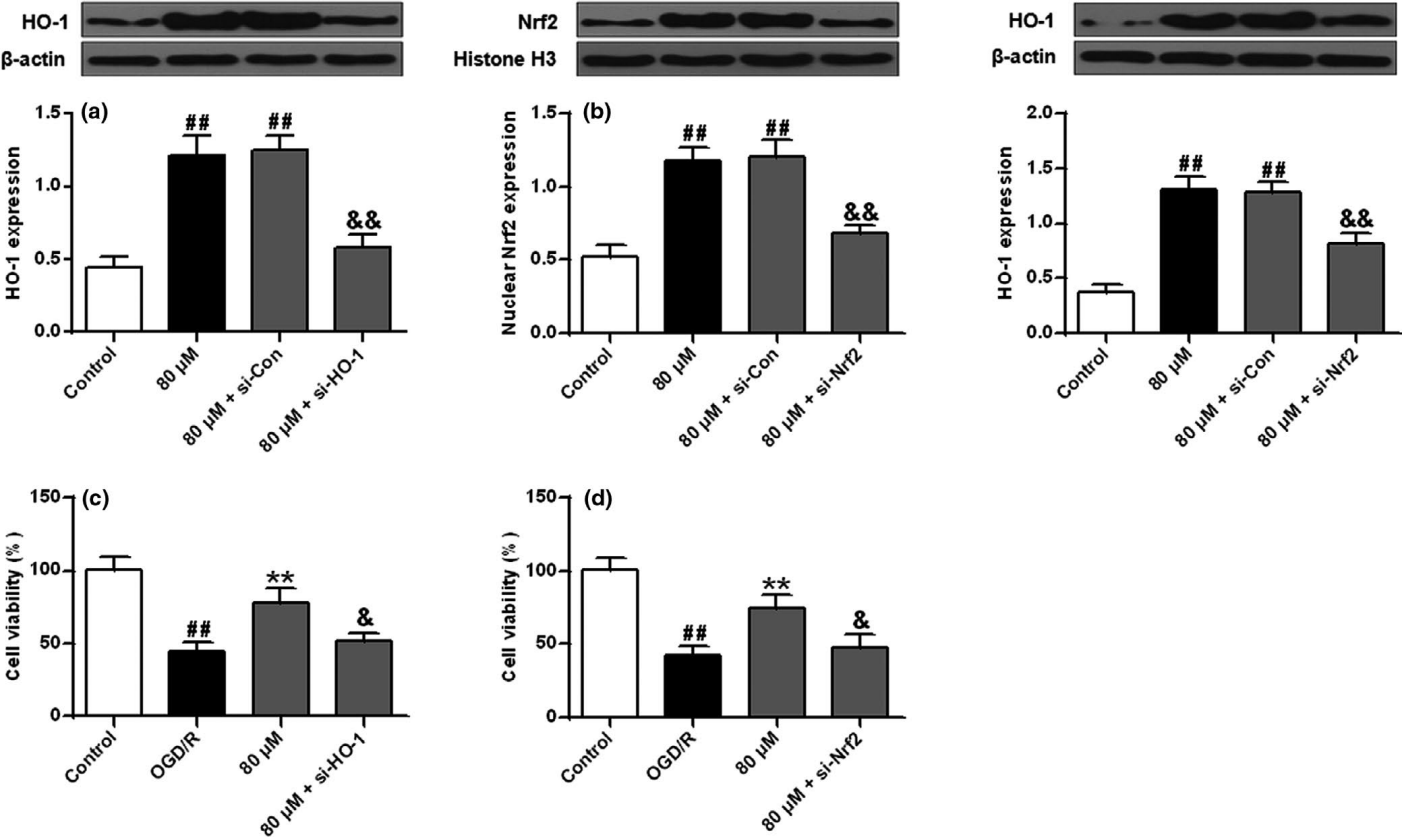
ISO produced neuroprotection via the Nrf2/HO‐1 signaling pathway. (a, b) Protein expression levels of HO‐1 and Nrf2. *n* = 6. ^##^ *p* < .01 versus the control group. ^&&^ *p* < .01 versus the 80 μM+ si‐Con group. (C, D) Cell viability. *n* = 6. ^##^ *p* < .01 versus the control group; ** *p* < .01 versus the OGD/R group; ^&^ *p* < .05 versus the 80 μM group

siRNA was used to knockdown Nrf2 and HO‐1 expression. The results showed ISO significantly increased HO‐1 expression and Nrf2 nuclear translocation. Moreover, the former increase was reversed by the interaction of ISO with HO‐1 siRNA and Nrf2 siRNA (Figure 6a,b, *p* < .01), and the latter increase was reversed by the interaction of Nrf2 siRNA with ISO (*p* < .01) (Figure 6b). Cell viability analysis using MTT showed that cell viability was significantly reduced in the OGD/R group compared with the control group (*p* < .01) and ISO significantly increased cell viability compared with that in the OGD/R group (*p* < .01). The interaction of HO‐1 siRNA or Nrf2 siRNA with ISO reversed the effect of ISO on cell viability (*p* < .05) (Figure 6c,d).

### 3.7 PKCε‐mediated ISO‐induced neuroprotection, Nrf2 nuclear translocation and HO‐1 up‐regulation

**FIGURE 7:**
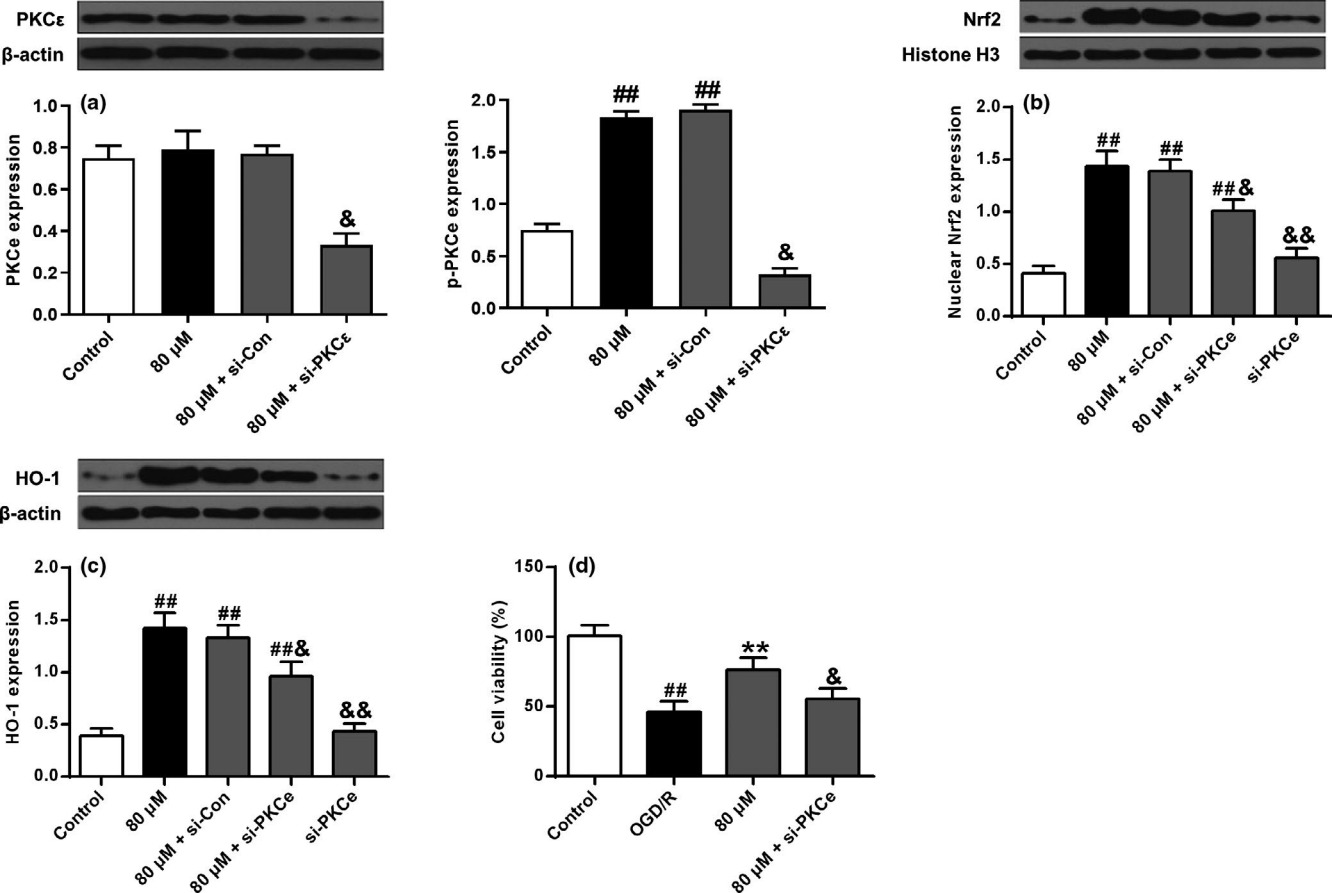
PKCε knockdown eliminated ISO‐induced neuroprotection. (a) PKCε and phosphorylated PKCε (p‐PKCε) levels, (b) Nrf2 expression, and (c) HO‐1 expression under different treatment conditions. *n* = 6. ^##^ *p* < .01 versus the control group; ^&^ *p* < .05, ^&&^ *p* < .01 versus the 80 μM + si‐Con group. (d) PKCε knockdown abolished ISO‐induced neuroprotection against OGD/R injury. *n* = 6. ^##^ *p* < .01 versus the control group; ** *p* < .01 versus the OGD/R group; ^&^ *p* < .05 versus the 80 μM group

As shown in Figure 7a, ISO treatment did not affect PKCε expression but increased PKCε phosphorylation, while PKCε siRNA significantly reduced PKCε expression and phosphorylated. Furthermore, as shown in Figure 7b,c ISO significantly increased Nrf2 nuclear translocation and HO‐1 expression. The interaction of PKCε siRNA and ISO reversed ISO effect on Nrf2 nuclear translocation and HO‐1 expression compared with ISO alone. PKCε siRNA alone had a more significant effect on Nrf2 nuclear translocation and HO‐1 expression (*p* < .01). Besides, compared with the control group, cell viability was significantly reduced in the OGD/R group (*p* < .01), and compared with the OGD/R group, ISO significantly increased cell viability (*p* < .01). The interaction of PKCε siRNA with ISO reversed ISO effect on cell viability (*p* < .05) (Figure 7d). These data indicated that ISO‐induced neuroprotection is mediated through activation of PKCε/Nrf2/HO‐1 signaling pathway.

## 4 DISCUSSION

Ischemic cerebrovascular disease is a frequently occurring disease in middle‐aged and older adults (He et al., 2017). Therefore, blood flow should be reconstructed by thrombolytic therapy within a practical time window after cerebral ischemic events to reduce brain damage. However, this could cause reperfusion injury, further aggravating brain tissue damage, resulting in more severe brain dysfunction (Carden, 2015). Cerebral ischemia‐reperfusion injury has increasingly become a significant cause of cerebral infarction and seriously threatened human physical and mental health (Liu, Zhang, et al., 2017). Currently, there is no effective intervention for cerebral ischemia‐reperfusion injury. Therefore, it is critical to identify new drugs to reduce cerebral ischemia‐reperfusion injury.

*Gnetum cleistostachyum* is a traditional Chinese medicine (Yao et al., 2010). Researches have shown that *Gnetum cleistostachyum* could augment immunity, enhance blood formation, improve blood circulation, and be an ideal substitute for ginseng (Yao et al., 2010). ISO extracted from *Gnetum cleistostachyum* has various biological activities, especially anti‐tumor activity, and is attracting more and more attention (Lei et al., 2015). Studies have demonstrated that ISO inhibits oxidized low‐density lipoprotein by reducing ROS production (Liu, 2004). ISO also possesses antioxidant activity and reduces cardiac hypertrophy by inhibiting Akt‐GSK3/p70S6K pathway (Wu et al., 2018). However, the molecular mechanism underlying ISO’s neuroprotection has not yet been fully elaborated.

The recovery of neurological function is the ultimate goal of stroke treatment (Liu, Wang, et al., 2017). In this study, ISO reduced the infarct volume and the behavioral score of experimental rats, significantly improved rats’ neurobehavioral function, and reduced the symptoms of neurological deficit. In vitro experiments showed that ISO significantly improved cell viability. These data indicated that ISO had protective functions against focal cerebral I/R injury.

Oxidative stress is one of the critical pathological mechanisms of ischemic stroke (Jena et al., 2017). Excessive ROS are mainly derived from mitochondria with a functional disorder, causing peroxidative damage of nucleic acids, lipids and proteins, and impairing normal physiological functions (Yang, Can, et al., 2017). Excessive ROS production promotes pathological processes of neurodegenerative diseases (Zhao et al., 2015). ROS can produce 8‐OHdG and 4‐HNE, which cause changes in cell function and eventually cause tissue and organ dysfunction (Ge et al., 2017). Therefore, 8‐OHd G, 4‐HNE, and NTY contents represent oxidative damage severity in cells (Sun et al., 2018). Our results showed that ROS production and 4‐HNE and 8‐OHdG content in the I/R group increased significantly, but significantly reduced after ISO treatment. These data suggested that cerebral ischemia and reperfusion increased free radical production in brain tissue, resulting in oxidative damage, and ISO inhibited free radicals production, thereby reducing oxidative brain damage by I/R.

Recent studies have shown that many active ingredients play a neuroprotective role via Nrf2 (Kou et al., 2015). HO‐1 reduces oxidative stress by regulating cytoprotective enzymes (Hong et al., 2017), while Nrf2 regulates the antioxidant response element of antioxidant enzyme ARE (Gong et al., 2017). Our study indicated that Nrf2 and HO‐1 expression in the nucleus was increased and the interaction of HO‐1 siRNA, Nrf2 siRNA and ISO reversed the effect of ISO on the cell viability, Nrf2 nuclear translocation and HO‐1 expression (*p* <.01).

Protein kinase C (PKC) is a threonine/serine protein kinase and a phospholipid‐ and Ca^2+^‐dependent protein kinase (Isakov, 2017). PKC**ε** belongs to the classical PKC family. It directly phosphorylates Nrf2 to separate Nrf2 from the INrf2‐Nrf2 complex and induces Nrf2 translocating from cytosol to nucleus (Chopra et al., 2018). This study showed that PKCε siRNA significantly affected Nrf2 and HO‐1 protein expression and reversed ISO’s effect on cell viability, Nrf2 nuclear translocation, and HO‐1 protein expression. These results indicated that ISO‐induced neuroprotection depended on increased PKCε expression and was associated with activation of the PKCε‐mediated Nrf2/HO‐1 signaling pathway.

A recent study has reported that ISO attenuated cerebral I/R injuries in rats by regulating PI3K/Akt signaling pathway (Sun & Cui, 2020). Our study revealed that ISO alleviated cerebral I/R injuries by inhibiting oxidative stress via regulating the PKCε/Nrf2/HO‐1 pathway, suggesting that ISO protects against cerebral I/R injuries by modulating multiple pathways. A previous study has reported that ISO could scavenge two free radicals (NO and NO_2_) (Zhu, et al., 2017), suggesting it may also protect against cerebral I/R injuries by inhibiting nitrosative stress, a critical player in the pathological process of brain ischemia (Tao et al., 2013; Zhao et al., 2018). However, due to limited funding, the hypothesis was not explored in this study. Besides, our study only investigated the effect of ISO on the neuron. Whether ISO affects other types of cells in the brain remains unclear. Other possible molecular mechanisms underlying ISO’s protective effects on cerebral I/R injury will be explored in the future.

## 5 CONCLUSION

ISO attenuated cerebral I/R injuries in rats by activating the PKCε/Nrf2/HO‐1 signaling pathway. As a survival signaling pathway, PKCε/Nrf2/HO‐1 signaling pathway may become a therapeutic target for ischemic cerebrovascular diseases in the future.

## CONFLICT OF INTEREST

The authors declare no competing interests.

## AUTHOR CONTRIBUTION

ZS and BX supervised the study. ZX, KZ, CW, BY and DK collected the experimental data and prepared the manuscript.

## ETHICAL APPROVAL

All applicable national and institutional guidelines for the Care and Use of Animals were followed. Animal experiments were approved by the Animal Ethics Committee of Chinese PLA General Hospital following the guidelines for the Care and Use of Laboratory Animals. This study passed the ethical reviewer of Chinese PLA General Hospital.

## DECLARATIONS

Not applicable.

## INFORMED CONSENT

No formal consent is required.

## CONSENT FOR PUBLICATION

Not applicable.

### PEER REVIEW

The peer review history for this article is available at https://publons.com/publon/10.1002/brb3.2143.

## Supporting information

Fig S1

## Data Availability

The analyzed data sets generated during the study are available from the corresponding author on reasonable request.
