# Supplementary figures and images for: Isorhapontigenin ameliorates cerebral ischemia/reperfusion injury via modulating Kinase Cε/Nrf2/HO‐1 signaling pathway
Source: Brain Behav. 2021 Jun 8;11(7):e02143. doi: 10.1002/brb3.2143 (PMC8323036; doi:10.1002/brb3.2143)

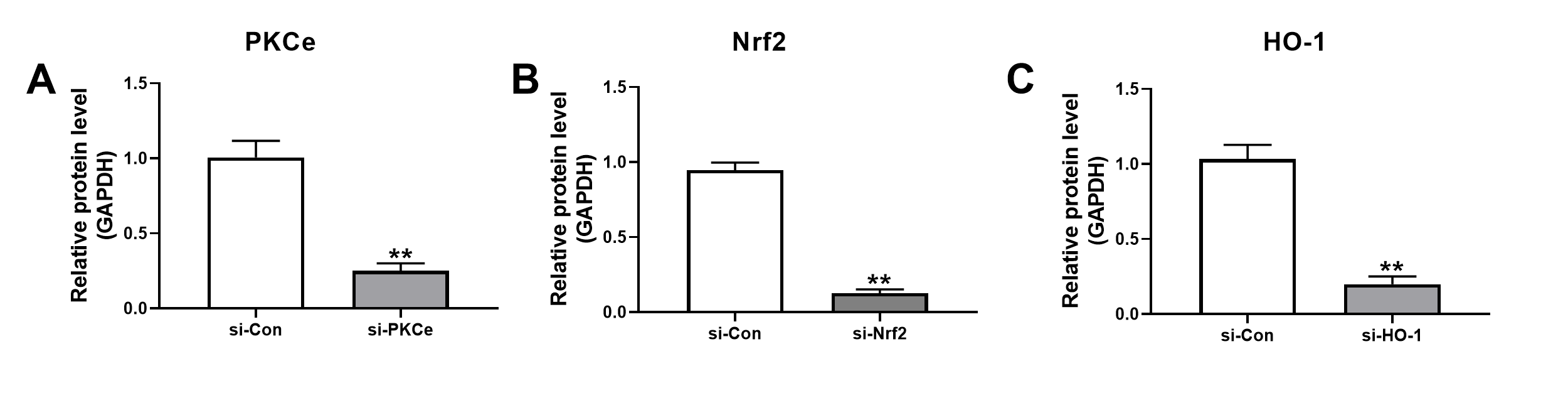

Supplement: Supplementary file 1 — Fig S1 [file BRB3-11-e02143-s001.tif]
